# Use of tramadol as analgesic alternative in Harris hawk (*Parabuteo unicinctus*)

**DOI:** 10.1002/vms3.1304

**Published:** 2023-10-25

**Authors:** Carlos Leonel Hernández‐Millán, Teódulo Quezada Tristán, Raúl Ortiz Martínez, Valdivia Flores Gerardo, Martínez‐Haro Marcela, Jaramillo Juárez Fernando

**Affiliations:** ^1^ Veterinary Sciences Department Universidad Autónoma de Aguascalientes Aguascalientes Aguascalientes Mexico; ^2^ Soil and Water Department Universidad Autónoma del Estado de México Touca Estado de México Mexico

**Keywords:** analgesia, Harris hawk, pain, sedation, tramadol

## Abstract

**Background:**

The Harris hawk is a bird of prey susceptible to traumatic injuries because it is useful for several purposes such as conservancy, biological control and falconry. Once received in rehabilitation centres or specialized clinics, it is necessary to provide proper analgesia.

**Objectives:**

The aim of this study is to demonstrate the analgesic efficacy of tramadol in Harris hawks (PISADOL 50 PiSA Agropecuaria, S.A. de C.V. Calle 1 Norte, Manzana 2‐25 Parque Industrial Tula Atitalaquia, Hgo, México), by the assessment of nociceptive threshold.

**Methods:**

A total of 24 adult Harris hawks were selected from a rehabilitation centre. The birds were randomly divided into four groups: control (saline solution), 5.0, 15.0 and 30.0 mg/kg of intramuscular tramadol. Nociception was produced with electrical stimuli of 9 V, applied in propatagial skin at 1, 5, 10, 20, 30, 45, 60, 90, 120, 180, 240, 300 and 360 min, assessing the nociceptive threshold and sedative effects produced by each treatment.

**Results:**

No difference was observed between control and tramadol group 5 mg/kg. At 15 mg/kg, the pain threshold increased from 20 to 240 min, with minimal sedative effects. At 30 mg/kg, there was a marked increase in pain threshold from 10 to 300 min, and sedative effects like wing and head drooping for a period of 90 min.

**Conclusions:**

Tramadol can be an analgesic alternative for Harris's hawks, as it decreases the response to painful stimuli in this species when administered by intramuscular route.

## Graphical abstract

Tramadol is an analgesic alternative for Harris's hawks, as it decreases the nociceptive response when administered by intramuscular route; in this study, were used 24 birds randomly divided into four groups: control (saline solution), 5.0, 15.0 and 30.0 mg/kg. An electrical stimulus of 9 V was applied on skin surface, and nociception was measured. The nociceptive response decreases significantly from 15 to 30 mg/kg. Sedative effects were observed in 30 mg/kg group.

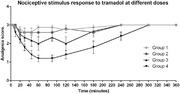

## 1 INTRODUCTION

The Harris's hawk is a bird of prey classified as abundant in most countries of America (Sibley, 2014). It spreads from the south of the USA to the south margins of Argentina (Frost, 2007; Howell & Webb, 1995). Due to its social behaviour and good adaption to captivity, it has been the most common bird of prey in private collections, breeding centres, zoos, rehabilitation centres and bird control services around the world. This fact makes that species one of the most susceptible to traumatic injuries associated to management in captivity, or the risks of its wild environment (Sheelings, 2014). For example, bone fractures due to trauma, and other diseases, involve painful stimulus. Once those birds are received in rehabilitation centres or specialized veterinarians, proper pain control treatment is necessary (Geelen et al., 2013). There is limited research available in relation to pain management in this avian species (Paul‐Murphy & Hawkins, 2012).

Opioids have been documented as one of the best therapeutic tools for pain control due to they are relatively safe and effective (Grond & Sablotzki 2004). The respiratory side effects may be reversed by the use of other drugs like naloxone and doxapram (Plumb, 2008). Opioids may be administered prior to bird surgery without renal or coagulative alteration, as seen with non‐steroidal anti‐inflammatory drugs (NSAIDs) (Souza et al., 2010).

Tramadol is an alternative to produce analgesia in birds of prey; nevertheless, it has not been sufficiently studied (Malik & Valentine, 2018; Tranquilli et al., 2007). Tramadol is a synthetic analogue of codeine, with central action as weak agonist of opioid of mu receptor. Part of its analgesic effect is produced by the inhibition of norepinephrine and serotonin reuptake at the synaptic cleft (Minami et al., 2007; Tranquilli et al., 2007). Affinity of tramadol for mu opioid receptor is 1/6000 compared with morphine. The positive enantiomer binds to the mu receptor and inhibits serotonin reuptake. The negative enantiomer produces a stimulation of adrenergic alpha 2 receptors and inhibits the recapture of norepinephrine (Ide et al., 2006; Lewis & Han, 1997).

The complementary and synergic actions of both enantiomers improve the analgesic efficacy, enhancing the inhibitory effects over transmission pathways of pain in spinal cord (Grond & Sablotzki, 2004). Their effects are antagonized primarily by naloxone, a pure antagonist of mu, kappa and sigma opioid receptors, but mainly for mu receptor (Plumb, 2008). Tramadol is an opioid family analgesic and has particular features useful in pain control and neuropathic pain in a wide range of animal species (Shaver et al., 2009). Sheelings (2014) used tramadol at doses of 5–30 mg/kg intramuscularly with favourable results in several species of birds including birds of prey. Moreover, Souza et al. (2009) used Red‐tailed hawks (*Buteo jamaicensis*) at doses of 11 mg/kg orally and 4 mg/kg intravenously, as well as Sanchez‐Migallón et al. (2014) used American kestrels (*Falco sparverius*) at doses of 5, 15 and 30 mg/kg orally. A therapeutic margin has not been established in birds of prey at this point and does not exist pharmacokinetic and pharmacodynamics studies that support the dosage in this species, because of that a more detailed research is necessary about safety and efficacy of tramadol in Harris hawks.

The pain has two kinds of nerve fibres (type Aδ and C fibres) that differ in conduction velocity and sensitive localization. The methods used most recently to evaluate the pain threshold consist in measuring the thermal nociception, by thermal stimulus using heat plates with a range of temperature from 27.0 to 55.0°C (Gustavsen et al., 2009). This is an adaption of heat plate used for the pain evaluation of different analgesic drugs in domestic mammals (Benítez et al., 2015).

In birds, exist several types of mechanical stimuli as finger or skin twitch, and feather plucking, to evaluate pain response. Thermal and mechanical stimuli have been used for opioid analgesia evaluation; however, the response to this type of stimulus may differ among individuals, sex and age. An electrical quantifiable stimulus induces more predictable responses and has been used in other studies in mammals (Desmarchelier et al., 2012). Tawfik et al. (2009) reported a clinical evaluation of tramadol in chicks, by the use of electrical stimuli of 9 V in propatagial skin, and the administration of tramadol at different doses to identify both nociceptive response to electrical stimuli, and sedation signs produced by tramadol in this species, demonstrating its analgesic and adverse effects.

The objectives of this study were to identify the differences in electrical antinociceptive effects of tramadol in Harris hawks, produced by electrical stimulus of 9 V applied on propatagial skin, with different doses of tramadol administered by intramuscular route, and evaluate the presence of adverse effects.

## 2 MATERIALS AND METHODS

A total of 24 adult Harris hawks, 9 males and 15 females in a weight range from 700 to 1100 g, were obtained from 2 rehabilitation centres in central México. In physical exams, hematologic evaluation of red blood cells, white blood cells, haematocrit, mean corpuscular haemoglobin value, blood chemistry, alanine aminotransferase, aspartate aminotransferase, total protein, glucose, blood urea nitrogen, creatinine, alkaline phosphatase and parasitological studies were performed in each bird. Once verified absence of preexistent pathologies, the birds were included in this study. All conditions established by the University Ethics Committee for Animal Use in Teaching and Research were applied.

Four groups with six animals were selected randomly, and one treatment to each group was assigned to evaluate nociceptive threshold after tramadol administration by intramuscular route. Groups were as follows: group 1 (control, 0.9% saline solution IM), group 2 (5 mg/kg), group 3 (15 mg/kg) and group 4 (30 mg/kg). Once calculated tramadol dose, an electrical bio stimulator was attached to the surface of propatagial skin to induce a nociceptive stimulus. Nociceptive responses were evaluated at 1, 5, 10, 20, 30, 45, 60, 90, 120, 180, 240, 300 and 360 min after administration (MAA), and observations were recorded according to Table 1.

**TABLE 1:** Analgesic score.

Score assigned	Level of nociceptive response
1	Low response
2	Mid response
3	High response

*Note*: 1: Wing movement in rest posture. 2: Wing movement, open wings, defensive posture. 3: Wing movement, open wings, defensive posture, vocalization.

*Source*: Adapted from Tawfik et al. (2009).

Additionally, sedation response induced by tramadol was evaluated and registered using a score designed for chicks by Tawfik et al. (2009), and a numeric value was assigned to each observation according to Table 2.

**TABLE 2:** Sedation score.

Score	Sedative effects
0	Normal
1	Wing droop
2	Head droop
3	Ataxia
4	Recumbency

*Note*: 0: Normal posture, head in alert posture, perched on both legs. 1: Wing tip droop, with wing tip touching the perch and tail feathers. 2: Head droop, includes cervical flexion. 3: Bird unable to maintain a posture, still perching on both legs but also with the wings and tail. 4: Unconscious bird, unable to perch (Tawfik et al., 2009).

## 3 STATISTICAL ANALYSIS

The results were analysed with non‐parametric test; Shapiro–Wilk and Kolmogorov–Smirnoff tests were applied to determine the normality of data and Levene and Durbin–Watson test for the homogeneity of the variance and data independence.

## 4 RESULTS

Results are expressed as mean ± standard error, with a significance level of *p* < 0.05 (Statistical Analysis System [SAS], 1999).

**FIGURE 1:**
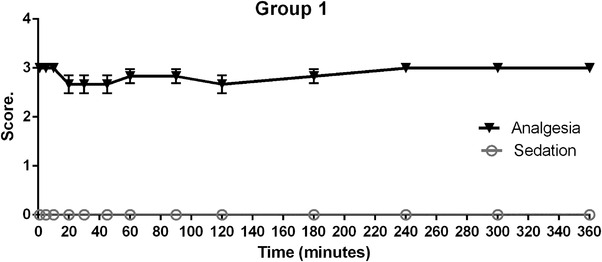
Group 1 (saline solution) results, analgesia (solid black line triangles) and sedation (grey line circles) in Harris hawks (*n* = 6). Showing mean ± SE after intramuscular administration of saline solution.

Figure 1 shows Harris hawks in group 1 which received saline solution, observing that analgesia was maintained in level 3 (intense response to stimulus) according to sedation score. With no significant variations from 20 to 240 min (*p* > 0.05), sedation effects were not observed.

**FIGURE 2:**
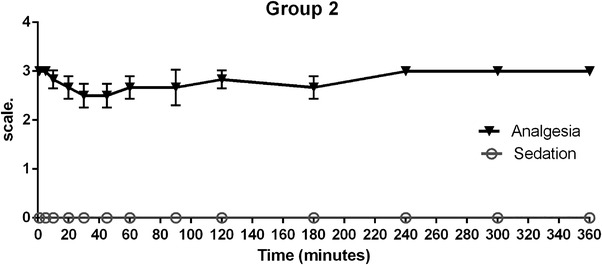
Results of group 2 analgesia (solid black line triangles) and sedation (grey line circles) in Harris hawks (*n* = 6). Showing mean ± SE bars after intramuscular administration of 5 mg/kg of tramadol.

The animals in group 2 (5 mg/kg of tramadol, intramuscular) showed a decrease in nociceptive response between 30 and 45 min after drug administration and returned to normal levels or intense response in the analgesia score until the end of observation period (Figure 2). Sedation adverse effects were not observed in this group (*p* > 0.05).

**FIGURE 3:**
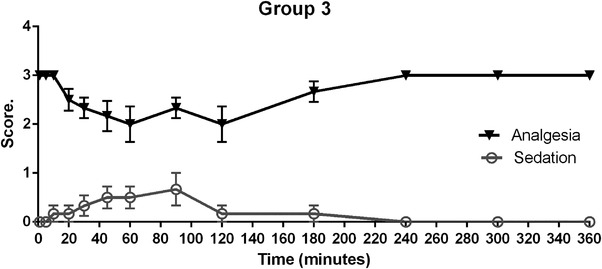
Results of group 3 analgesia (solid black line triangles) and sedation (grey line circles) in Harris hawks (*n* = 6). Showing mean ± SE bars after intramuscular administration of 15 mg/kg of tramadol.

Results of group 3 of birds that received a dose of 15 mg/kg are shown in Figure 3 where nociceptive response significantly decreases from 10 min after tramadol administration, and rising and maximum levels of analgesia were obtained at 60 min and maintained for 1 h to finally decrease at 240 min (*p* < 0.05). Sedation effects were observed in this group starting at 10 min after tramadol administration, rising to maximum levels at 90 min with level 1 of sedation score (wing tip droop) and finally decreasing at 240 min, with no more signs of sedation or analgesia until the end of the observation period.

**FIGURE 4:**
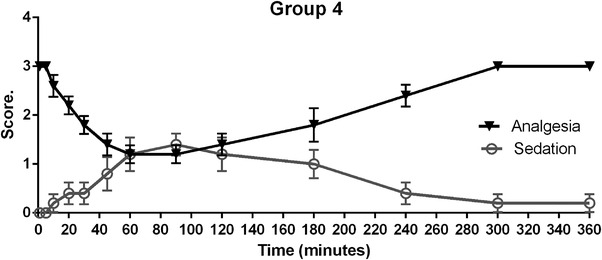
Results of group 4 analgesia (solid black line triangles) and sedation (grey line circles) in Harris hawks (*n* = 6). Showing mean ± SE bars after intramuscular administration of 30 mg/kg of tramadol.

In relation to animals treated with tramadol (30 mg/kg, intramuscular), Figure 4 shows that, 10 min after drug administration, nociceptive response decreases with a maximum effect of analgesia from 60 to 90 min at level 1 (minimal response). The analgesic effect begins to decrease at 120 min and finally ends at 300 min. Sedation effects were observed from 10 min at level 2 (head droop) with maximum effects at 90 min and decrease at level 1 (wing droop), and it maintains until the end of observation period.

**FIGURE 5:**
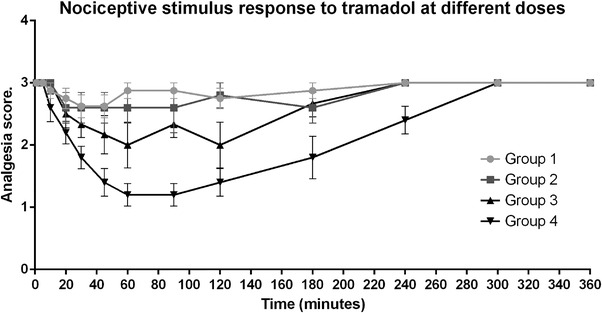
Results of all groups in analgesia in Harris hawks (*n* = 24). Showing mean ± SE bars after intramuscular administration in four treatments. Group 1 (saline solution, clear grey line circles), group 2 (5 mg/kg of tramadol, grey line squares), group 3 (15 mg/kg of tramadol, black line up arrows) and group 4 (30 mg/kg of tramadol, black line down arrows).

Finally, Figure 5 shows a comparison of analgesic results (mean ± SE) of all groups. The groups 1 and 2 show that response to nociceptive stimulus was intense during the observation period, with no significant differences. However, in group 3, the response to nociceptive stimulus decreases at a moderate level for a 60 min period (from 60 to 120 min after tramadol administration). Additionally, the group 4 shows a lower response to nociceptive stimulus for a period of 90 min, maintaining in level 1 of lower response and a total duration of analgesic period of 290 min, with maximum effect between 69 and 90 min after drug administration.

## 5 DISCUSSION

Pain is an unpleasant sensation occurring majority due to tissue damage, and it is classified into three types: (1) nociceptive pain, produced by tissue injury, (2) neuropathic pain, due to nerve injury and (3) neuroplastic pain, due to musculoskeletal disease like inflammatory pain. Various pharmacological approaches have been used for its treatment with drugs such as paracetamol, NSAID category drugs, anti‐cytokines and opioids. Tramadol is used for the treatment of pain and possesses potent analgesic activity (Bravo et al., 2017; Dayer et al, 1997; Subedi et al, 2019).

Evaluation of antinociceptive effects of opioids in birds, minimal invasion methodologies, has been used as warm perches for stimulating Aδ and C type of nociceptive nervous fibres. Nevertheless, the use of thermoception for analgesic evaluation is questionable due to the individual variability showed in some studies as Sanchez‐Migallón et al. (2012), which reported the influence of age and sex in the response to thermal stimulus. An electrical quantifiable stimulus induces more predictable responses and has been used in other studies in mammals. The electrical method that stimulates Aδ fibres was used in this study and caused uniform responses in all 24 birds, without differences between sex and age. It is important to note that the voltage capable to produce an adequate stimulus was 9 V in concordance with reported by Tawfik et al. (2009) who used chicks.

Group 1 showed a particular response to electrical stimulation because there was a diminution of nociceptive response for a period of 40 min and returned to normal levels at the end of study. This may probably be due to the activation of descending pain pathways and to the release of endogenous opioids, as reported by Sousa and Asmawi (2015), who observed that control group showed an analgesic‐like response. However, the in vitro hydrolysis of (leu)enkephalin is more rapid in chick plasma (half‐life, 0.7–1 min) than in rat (half‐life, 2–2.5 min) or mouse (half‐life, 9–14 min) plasma (Shibanoki et al., 1991).

The results of group 2 (tramadol, 5 mg/kg) were similar to that observed in group 1: The nociceptive umbral increased during the first 90 min. Thus, these results are not significantly different from group 1. Inferior doses to 5 mg/kg has been reported as analgesic in several species, administered orally or intravenously, as reported in horses by Giorgi et al. (2006) and Guedes et al. (2014), as well as rhesus monkeys by Kelly et al. (2015). These tramadol doses differ from the doses administered to animals of this study.

At 15 mg/kg of tramadol doses, the animals showed an increase in nociceptive threshold and a longer analgesia period, starting at 10 min and maintained until 240 min after drug administration. Additionally, sedation effects were observed in a longer period than group 2, at the same time of analgesic period, rising to level 2 of response (wing droop). This dose decreased effectively nociceptive response as reported by Sanchez‐Migallon et al. (2014) in American Kestrels by oral route, evaluating foot withdrawal with thermal nociception. However, sedation effects were not reported in this species using 15 mg/kg doses.

The tramadol dose of 30 mg/kg produced a more marked decrease in nociceptive response with onset of effects at 5 min and until 300 min after drug injection. There was an inhibition of response to stimuli (level 1). However, the nociceptive perception was not completely abolished, only a great decrease was observed, even in birds with sedative effects. Initially, the sedative effects of this group were marked (level 3, head droop) and persisted at level 1 until the end of study.

Only the group 4 (tramadol, 30 mg/kg) showed marked sedative effects, but birds treated with lower doses of tramadol developed only minimal signs. Sanchez‐Migallon (2012), with American kestrels and Shellings (2009), with several species of birds of prey have not reported marked sedation signs in doses of tramadol of 30 mg/kg, administered orally. In our study, additional adverse effects as vomiting or diarrhoea were not observed.

We demonstrated that tramadol improves a decrease of nociception in a dose‐dependent way, from 10 to 300 min after intramuscular administration. In addition, the results of this study showed that tramadol doses to produce analgesic effects in Harris hawks are between 5 and 30 mg/kg of weight. Depending on therapeutic objective, it is possible to administer tramadol intramuscularly at the mentioned doses. Mean dose of tramadol (15 mg/kg IM) produces analgesia from 10 to 240 min. Tramadol doses less than 5 mg/kg do not appear to be effective in producing analgesia in this species.

Finally, the dose range of tramadol to produce optimal analgesia in this species is between 15 and 30 mg/kg. On the other hand, the sedative effects occurred with doses greater than 15 mg/kg and increased proportionally to the administered dose, until the sign of head drooping was observed at 30 mg/kg.

In conclusion, tramadol can be an analgesic alternative for Harris's hawks, as it decreases the response to painful stimuli in this species when administered by intramuscular route.

## AUTHOR CONTRIBUTIONS

Teódulo Quezada Tristán and Jaramillo Juárez Fernando with their wide contribution in methodological and experimental design as much as revisions; Martínez‐Haro Marcela with his collaboration in clinical work and methodology application; Valdivia Flores Gerardo with statistics design of study; Raúl Ortiz Martínez with results analysis and interpretation; and Carlos Leonel Hernández‐Millán with the work development, bird's management and redaction.

## CONFLICT OF INTEREST STATEMENT

Authors declare that they have no conflicts of interest.

### PEER REVIEW

The peer review history for this article is available at https://www.webofscience.com/api/gateway/wos/peer‐review/10.1002/vms3.1304.

## ETHICS STATEMENT

The authors confirm that the ethical policies of the journal, as noted on the journal's author guidelines page, have been adhered to and the Ethics Committee for Animal use in Teaching and Research of Autonomous University of Aguascalientes approval has been received. The authors confirm that they have adhered to either US standards for the protection of animals used for scientific purposes, and thus by Norma Oficial Mexicana (NOM‐062‐ZOO‐1999), Especificaciones técnicas para la producción, cuidado y uso de los animales de laboratorio.

## Data Availability

The data that support the findings of this study are available from the corresponding author upon reasonable request.
